# Changes in Melanoma Cell Morphology Following Inhibition of Cell Invasion by Third-Generation mTOR Kinase Inhibitors

**DOI:** 10.3390/ijms26167770

**Published:** 2025-08-12

**Authors:** Dorota Ciołczyk-Wierzbicka, Martyna Sikorska-Duplicka, Marta Zarzycka, Grzegorz Zemanek, Karol Wierzbicki

**Affiliations:** 1 Center for Medical Genomics—OMICRON, Jagiellonian University Medical College, ul. Kopernika 7c, 31-034 Kraków, Poland; 2 John Paul II Hospital, ul. Prądnicka 80, 31-202 Kraków, Poland; martyna.duplicka@gmail.com (M.S.-D.); karol.wierzbicki@uj.edu.pl (K.W.); 3 Chair of Medical Biochemistry, Jagiellonian University Medical College, ul. Kopernika 7, 31-034 Kraków, Poland; marta.zarzycka@uj.edu.pl (M.Z.); grzegorz.zemanek@uj.edu.pl (G.Z.); 4 Department of Cardiovascular Surgery and Transplantology, Institute of Cardiology, Jagiellonian University Medical College, ul. Prądnicka 80, 31-202 Kraków, Poland

**Keywords:** cell invasion, mTOR protein kinase inhibitors, melanoma, cell morphology

## Abstract

Melanoma is one of the most invasive skin cancers with the highest mortality risk. The PI3K/AKT/mTOR signaling pathways are a key regulatory point related to growth factors and involved in the cell’s energy metabolism. They are responsible for cell life processes such as growth, proliferation, invasion, survival, apoptosis, autophagy, and angiogenesis. The studies undertaken concerned the effect of protein kinase inhibitors involved in the signaling pathways of AKT, MEK, and mTOR kinases on the expression of cytoskeletal and extracellular matrix proteins, invasion process, and activities of the matrix metalloproteinases (MMPs): MMP-2 and MMP-9 in melanoma cells. The study used mTOR kinase inhibitors: Everolimus and Torkinib; dual PI3K/mTOR inhibitors BEZ-235 and Omipalisib; and the mTORC1/2 inhibitor OSI-027. These compounds were used both as monotherapy and in combination with the MEK1/2 inhibitor AS-703026. mTOR kinase inhibitors, especially the third generation in combination with the MEK 1/2 kinase inhibitor AS-703026, significantly inhibited invasion and metalloproteinases (MMPs) activity in melanoma cell lines. The inhibition of the cell invasion process was accompanied by a significant change in the expression of proteins associated with EMT. The morphology of cells also changed significantly: their thickness, volume, roughness, convexity of shape, and irregularity, which may be a good diagnostic and prognostic factor for the response to treatment. Our studies to date on the effect of three generations of mTOR kinase inhibitors on the inhibition of the invasion process, the activation of apoptosis, and the reduction in cell proliferation suggest that they may be an important target for anticancer therapy.

## 1. Introduction

Melanoma is among the most heterogeneous and treatment-resistant malignancies, with a steadily increasing global incidence over recent decades. Advanced-stage melanoma is associated with poor outcomes, and the five-year survival rate for patients diagnosed at stage IV remains as low as 23% [1]. Its progression reflects several emerging hallmarks of cancer, including phenotypic plasticity, evasion of immune destruction, and deregulated cellular energetics [2]. The intrinsic heterogeneity of melanoma, combined with its remarkable phenotypic plasticity, contributes to frequent therapeutic failure and metastasis [3]. Only select patient subgroups benefit from current systemic therapies, highlighting the need for more refined and targeted strategies.

Melanoma cells employ a spectrum of migration and invasion mechanisms, including both individual and collective motility [4]. One major challenge in targeting tumor invasion lies in the dynamic adaptability of cancer cells, which can switch their mode of migration in response to environmental and pharmacological stimulus, thereby evading anti-metastatic therapies [4].

A key driver of tumor progression is the constitutive activation of the mTOR (mechanistic target of rapamycin) signaling pathway, observed in multiple cancer types. mTOR dysregulation promotes cancer cell proliferation, angiogenesis, metabolic reprogramming, and survival while suppressing autophagy and apoptosis [5,6]. Rapamycin was the first mTOR inhibitor to receive FDA approval, initially for immunosuppression in transplant patients. Its success led to the development of more potent and selective mTOR-targeting compounds.

Traditional mTOR inhibitors such as Everolimus are allosteric inhibitors that bind to the FRB domain of mTOR. However, their incomplete inhibition of mTORC1 and lack of effect on mTORC2 limit their therapeutic scope [7]. ATP-competitive inhibitors such as OSI-027 and Torkinib target the kinase domain directly and suppress both mTORC1 and mTORC2. Dual PI3K/mTOR inhibitors—including BEZ-235 and Omipalisib—advance a step further by simultaneously targeting PI3K and mTOR kinases, exploiting their structural and functional interdependence.

One of the key pathways implicated in melanoma progression is the PI3K/AKT/mTOR signaling cascade, which plays a central role in regulating cell growth, proliferation, invasion, apoptosis, autophagy, and metabolic reprogramming [8,9,10]. This pathway is also closely linked with growth factor signaling and energy metabolism, making it a vital hub in tumorigenesis [11,12]. Dysregulation of mTOR contributes to enhanced survival and the invasive potential of melanoma cells, and it represents an attractive therapeutic target.

Despite the development of various mTOR inhibitors, the diversity of available compounds and their mechanistic nuances necessitate a comparative evaluation. In this study, we selected three mechanistically distinct generations of mTOR inhibitors—Everolimus (allosteric mTORC1 inhibitor), OSI-027 and Torkinib (ATP-competitive mTORC1/2 inhibitors), and BEZ-235 and Omipalisib (dual PI3K/mTOR inhibitors)—based on their documented efficacy and potential for combinatorial synergy [7,13].

Everolimus, a first-generation allosteric mTOR inhibitor, has demonstrated anti-proliferative activity in multiple malignancies, yet its therapeutic window is limited due to incomplete inhibition of mTORC1 and lack of activity against mTORC2. Moreover, studies indicate that Everolimus may exhibit complex, context-dependent, and dose-dependent effects on tumor cells [14], and its efficacy can be potentiated by metabolic modulators such as AICAR [15], underscoring the need for optimized combinations.

Tumor cell invasiveness involves dynamic remodeling of the cytoskeleton and extracellular matrix (ECM). Matrix metalloproteinases (MMPs), particularly MMP-2 and MMP-9, are crucial enzymes that mediate ECM degradation and promote tumor invasion and metastasis [16,17,18]. Therefore, investigating how mTOR inhibitors affect MMP activity and related phenotypic features such as morphology and motility is essential for understanding their anti-invasive potential.

In addition to traditional molecular endpoints, we also focused on morphological features such as cell thickness, volume, roughness, and shape parameters that may serve as quantitative markers of invasiveness and treatment response. Such phenotypic characteristics are increasingly recognized for their diagnostic and prognostic value. This approach aligns with recent optical profiling studies of melanocytic and melanoma cell lines, which emphasize the diagnostic value of quantitative phase metrics [19].

What distinguishes this study is its comparative evaluation of three mechanistically distinct generations of mTOR inhibitors, analyzed not only for their molecular effects but also for their impact on melanoma cell morphology, migration dynamics, and extracellular matrix remodeling. While prior studies have focused primarily on signaling endpoints, our approach integrates both molecular and phenotypic assessments.

Here, we investigated the effects of representative compounds from each class: Everolimus (allosteric mTORC1 inhibitor), Torkinib and OSI-027 (ATP-competitive mTORC1/2 inhibitors), and BEZ-235 and Omipalisib (dual PI3K/mTOR inhibitors). We further examined the synergistic effects of combining these agents with a MEK1/2 inhibitor (AS-703026) in highly invasive MEWO melanoma cells. Importantly, we also evaluated treatment-induced changes in morphological parameters—including cell thickness, volume, roughness, and shape irregularity—as potential markers of invasiveness and therapeutic response.

This multidimensional approach integrates molecular biology, live-cell imaging, and computational analysis to provide novel insights into how targeting the PI3K/AKT/mTOR and MEK pathways modulates melanoma cell behavior. Our results underscore the translational potential of combining targeted inhibitors to overcome resistance mechanisms and attenuate melanoma progression.

## 2. Results

### 2.1. Cytotoxicity Assay

No cytotoxic effects were observed following 24 h of treatment with AS-703026, Everolimus, BEZ-235, Omipalisib, OSI-027, or Torkinib. The lactate dehydrogenase (LDH) activity in the culture medium supplemented with 10% fetal bovine serum remained below 3.9%.

For a longer incubation time of 72 h, the combinations of inhibitors BEZ-235 with AS-703026 and Omipalisib with AS-703026 showed a cytotoxic effect of 9.3% and 11.8%, respectively. Slightly higher results were obtained for cells cultured in serum-free medium, but the cytotoxicity value never exceeded 4.3%. For the extended incubation time of up to 72 h, the cytotoxicity of the inhibitor combinations BEZ-235 with AS-703026 and Omipalisib with AS-703026 was also slightly higher and amounted to 10.3% and 12.4%, respectively.

For the melanoma cell line WM3211 (VGP), even after 72 h, no cytotoxic effect was observed. DMSO showed no cytotoxic effect at a concentration of approximately 0.1%), and the effect was also not observed at concentrations of 1–2%.

### 2.2. The Effect of mTOR Inhibitors on Melanoma Cell Morphology

We studied the effect of mTOR kinase inhibitors Everolimus, BEZ-235, and Omipalisib, alone and in combination with MEK1/2 kinase inhibitor AS703026, on cell morphological parameters after 72 h of stimulation. We determined parameters such as optical thickness, optical volume, roughness, shape convergence, and irregularity cells.

**Figure 1:**
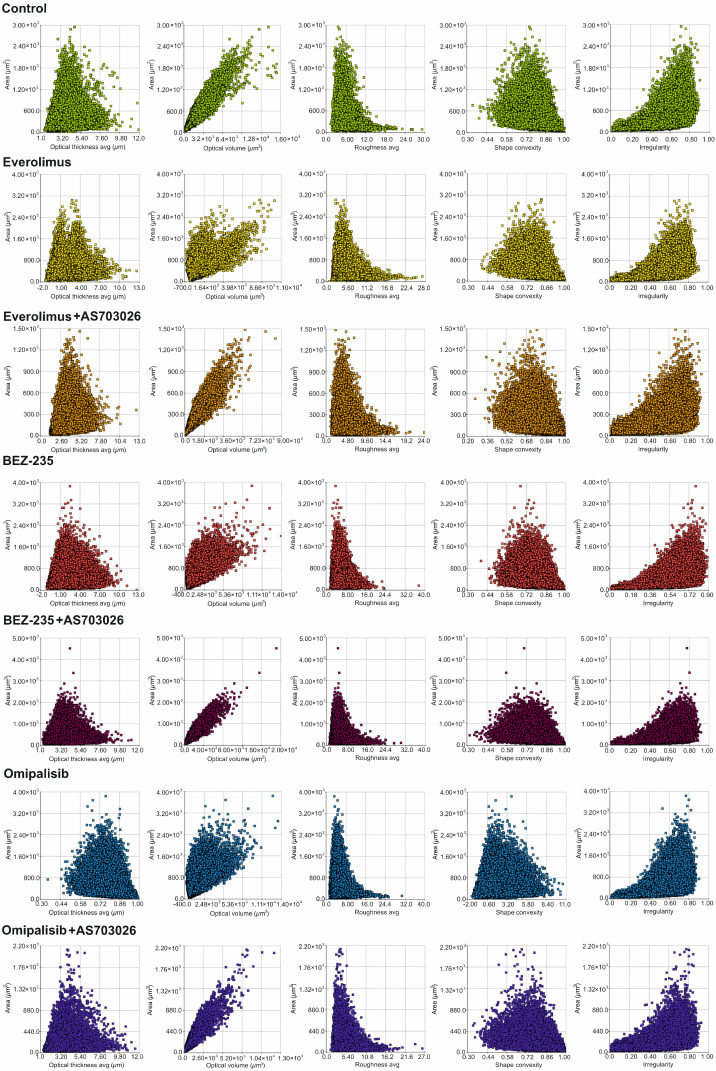
The effect of mTOR inhibitors on the module In-depth Analysis: “Cell Morphology”: optical thickness avg (µm), optical volume (µm^3^), roughness avg, shape convexity, irregularity per area (µm^2^) grown in MEWO melanoma cell line. The experiment was conducted for 72 h. For each inhibitor concentration tested, 178 consecutive time points were measured in 6 independent biological replicates.

**Figure 2:**
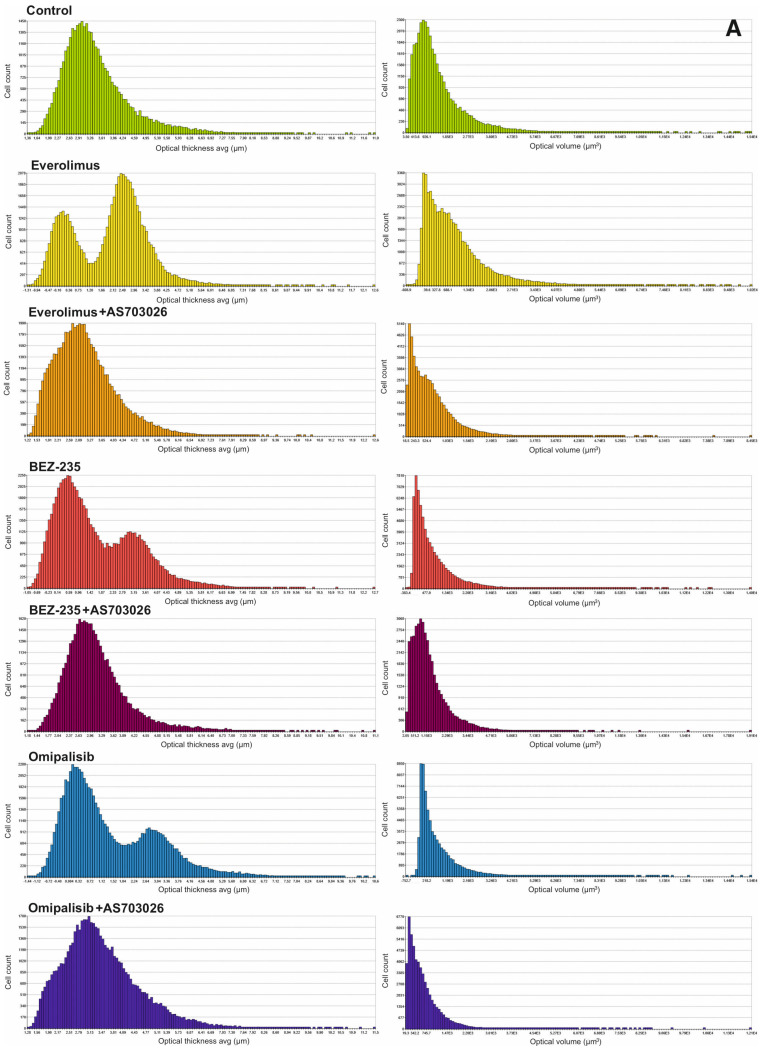

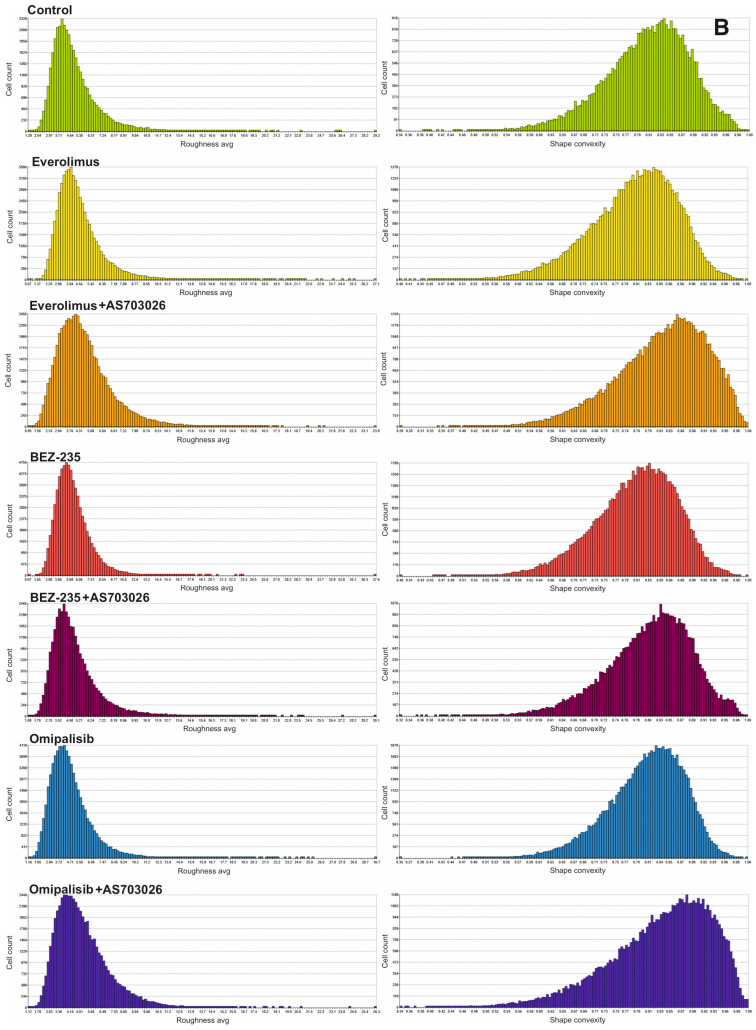

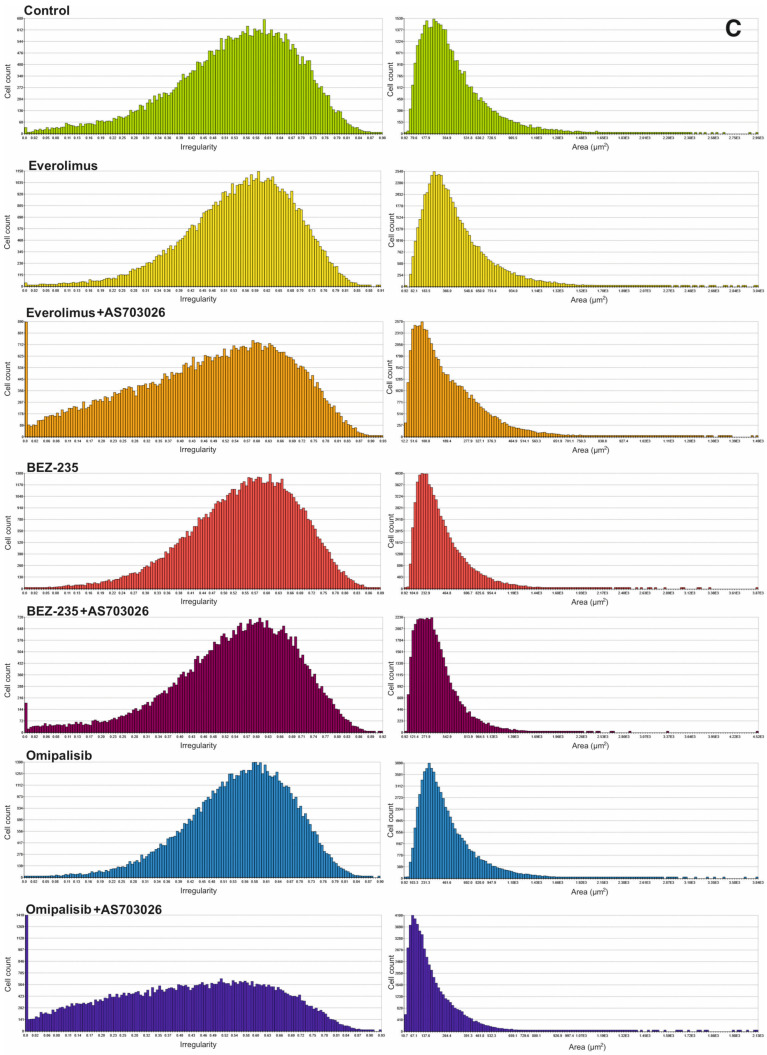
(**A**). The effect of mTOR inhibitors on the module In-depth Analysis: “Cell Morphology”: optical thickness avg (µm) in MEWO melanoma cell lines. The experiment was conducted for 72 h. For each inhibitor concentration tested, 178 consecutive time points were measured in 6 independent biological replicates (**B**). The effect of mTOR inhibitors on the module In-depth Analysis: “Cell Morphology”: roughness avg and shape convexity in MEWO melanoma cell lines. The experiment was conducted for 72 h. For each inhibitor concentration tested, 178 consecutive time points were measured in 6 independent biological replicates (**C**). The effect of mTOR inhibitors on the module In-depth Analysis: “Cell Morphology”: irregularity and area (µm^2^) in MEWO melanoma cell lines. The experiment was conducted for 72 h. For each inhibitor concentration tested, 178 consecutive time points were measured in 6 independent biological replicates.

The results obtained (Figure 1 and Figure 2) indicate a change in the morphological parameters of melanoma cells after treatment with mTOR kinase inhibitors. The greatest differences were observed in optical thickness, optical volume, and shape (Figure 1 and Figure 2, Table 1).

After the use of mTOR kinase inhibitors, a significant change in the thickness of MEWO melanoma cells was observed (Figure 2A, Table 1).

For Everolimus, BEZ-235, and Omipalisib inhibitors, the formation of two cell subpopulations was observed, shifting toward a decrease in melanoma cell thickness. The combination of mTOR inhibitors with the MEK1/2 kinase inhibitor AS703026 resulted in a clear shift toward a decrease in cell thickness, especially pronounced for the respective combinations of Everolimus and Omipalisib with AS703026.

A similar trend was observed for the optical volume of melanoma cells. In this case, the greatest effect was induced by the combination of Omipalisib and BEZ-235 with the MEK kinase inhibitor AS703026, respectively (Figure 2A, Table 1).

Melanoma cell roughness was also reduced after using all mTOR kinase inhibitors and their combination with the MEK1/2 kinase inhibitor AS703026 (Figure 2B, Table 1).

The highest shape uniformity and regularity were observed after using the BEZ-235 inhibitor alone and in combination with AS703026 (Figure 2B,C, Table 1).

### 2.3. Morphological Changes in the MEWO Melanoma Cell Line by Fluorescence Microscopy

**Figure 3:**
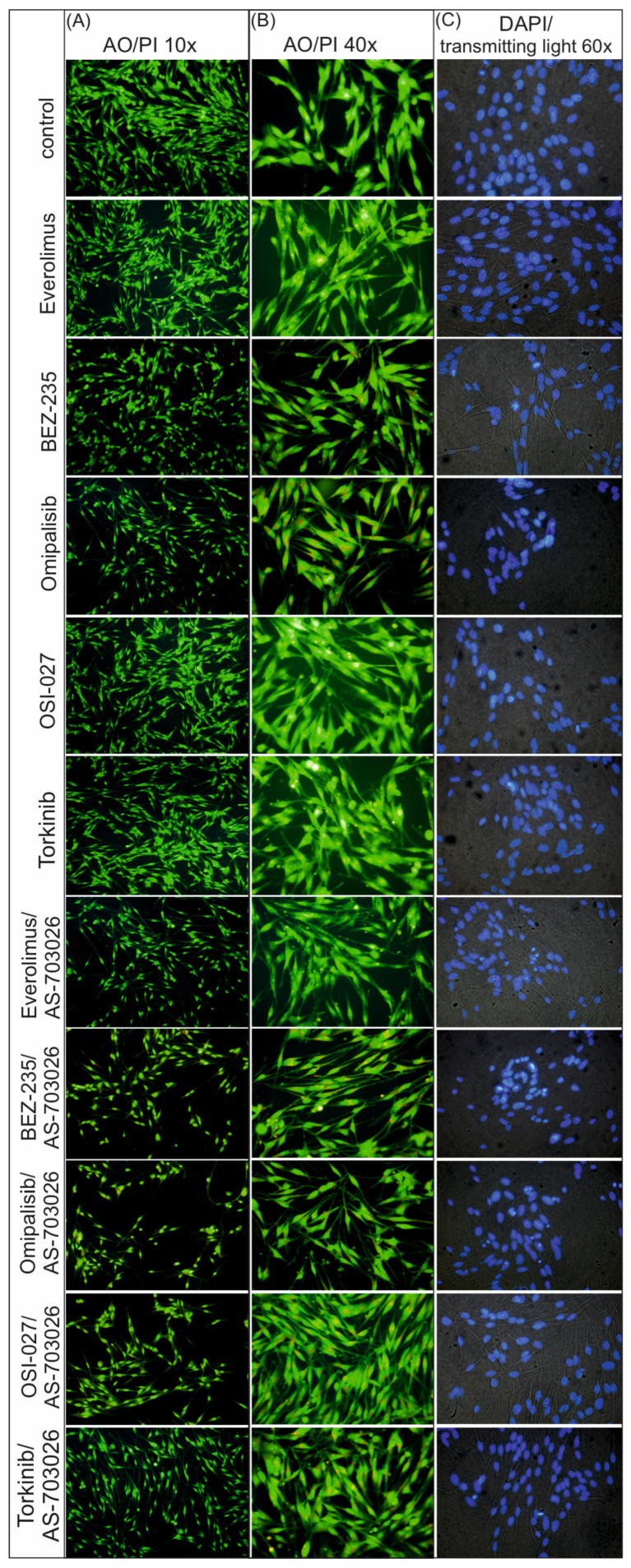
The morphological changes in MEWO melanoma cell lines after treatment with mTOR inhibitors for 24 h followed by (**A**) acridine orange/propidium iodide (AO/PI) microscope objective lens magnification 10×, (**B**) acridine orange/propidium iodide (AO/PI) magnification 40×, and (**C**) DAPI staining in the transmitted light magnification 60×. Details regarding the concentrations of the inhibitors are provided in the Materials and Methods section. The experiments were performed in triplicate.

The use of mTOR kinase inhibitors caused distinct changes in the cytoskeletal and nucleus structures. In control conditions, cells retained typical, diffuse morphology and uniform nuclear fluorescence (DAPI) without signs of chromatin condensation or fragmentation (Figure 3).

After the use of single mTOR inhibitors, an increased acridine orange staining of the cytoplasm was observed in the form of punctate orange structures, suggesting the accumulation of acidic cellular compartments, such as lysosomes or autophagosomes. This effect was most pronounced for dual AKT/mTOR pathway inhibitors (BEZ-235, Omipalisib), indicating a stronger activation of autophagy (Figure 3).

At the same time, DAPI staining revealed the presence of features typical of apoptosis in conditions with individual inhibitors, such as chromatin condensation and initial nuclear fragmentation.

As a result of treating melanoma cells with combinations of mTOR/PI3K inhibitors with AS-703026 (MEK1/2 inhibitor), significantly stronger morphological changes were observed. Cell nuclei showed clear fragmentation and strong chromatin condensation, which indicates advanced apoptosis. This was accompanied by the presence of numerous orange cytoplasmic structures after AO staining, which indicates simultaneous activation of autophagy (Figure 3).

The most pronounced changes were observed in the conditions with the combination of AS-703026 and Omipalisib and BEZ-235, which suggests a synergistic effect leading to intensive apoptotic–autophagic cell death (Figure 3).

### 2.4. The Effect of mTOR Inhibitors on the Melanoma Cell Invasion

**Figure 4:**
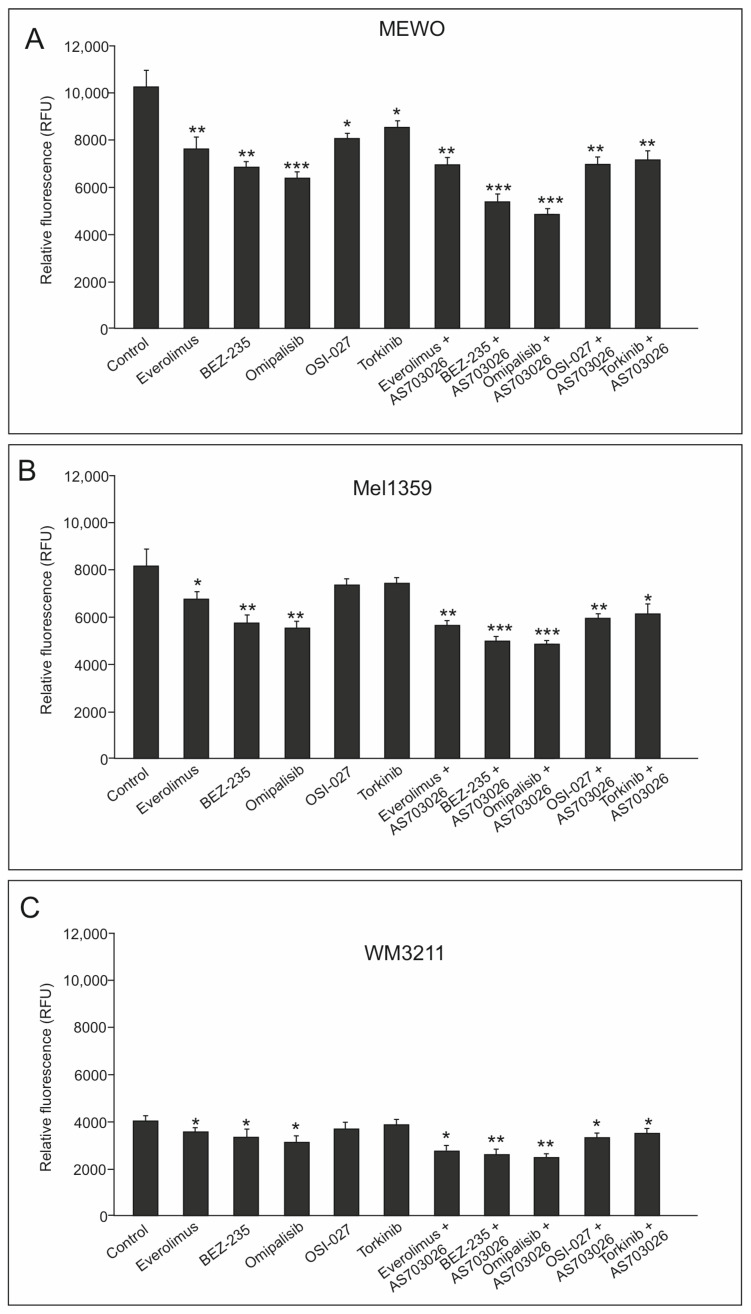
The effect of mTOR inhibitors on in vitro cell invasion melanoma cell lines: MEWO (**A**), Mel-1359 (**B**), and WM3211 (**C**). Cell invasive capacity was assessed via a Matrigel-coated Boyden chamber assay. The accompanying histogram presents the quantification of invasive cells. Data are expressed as the mean ± standard deviation from four technical replicates across two independent experiments. Statistical comparisons were performed using one-way ANOVA followed by Tukey’s post hoc test (Statistica version 12, StatSoft). Levels of statistical significance are denoted as follows: (*) *p* < 0.05; (**) *p* < 0.01; (***) *p* < 0.001. The primary melanoma cell line WM3211 (VGP) has the lowest sensitivity to the applied inhibitors, at 7–22% (Figure 4C).

Studies were performed using conventional Boyden trans-well methods. All tested melanoma cell lines, MEWO, Mel-1359, and WM3211, manifested the ability for cellular migration toward chemoattractant. The highest cellular invasion in vitro was shown by metastatic cell lines, MEWO, which was about 25% lower than Mel-1359. The primary line WMM3211 was characterized by about 60% lower invasiveness compared to the MEWO cell line (Figure 4A–C). These lines also showed a decrease in cellular invasion in vitro after using mTOR kinase inhibitors; the decrease ranged from 7% to 37% for the single inhibitors used (Figure 4A–C).

The largest decreases in in vitro invasion were observed for melanoma cells treated with dual PI3K and mTOR inhibitors: BEZ-235 and Omipalisib. For the inhibitor Omipalisib, the observed decreases were at the levels of 37% (*p* < 0.001) and 32% (*p* < 0.01) for the MEWO and Mel-1359 lines, respectively, while they were slightly lower for the BEZ-235: at 33% (*p* < 0.01) and 30% (*p* < 0.01), respectively (Figure 4A,B). A high reduction in invasion was observed with the mTOR inhibitor Everolimus at 25% (*p* < 0.01) for the MEWO cell line (Figure 4A,B).

Combining mTOR inhibitors with the MEK1/2 inhibitor AS-703026 resulted in much greater reductions in in vitro invasions, reaching up to 50% (Figure 4A–C).

The use of dual PI3K and mTOR inhibitors, BEZ-235, and Omipalisib, in combination with the MEK1/2 inhibitor AS-703026, gave a synergistic effect and a decrease in cell invasion by 47% (*p* < 0.001) for the BEZ-235 inhibitor. The highest efficiency was 52% (*p* < 0.001) for the Omipalisib inhibitor for the MEWO cell line (Figure 4A). Other mTOR inhibitors, together with the MEK1/2 inhibitor AS-703026, also showed an apparent effect. The mTOR inhibitor Everolimus, in combination with the MEK1/2 inhibitor AS-703026, generated a decrease in invasion by approximately 30% (*p* < 0.01) (Figure 4A).

### 2.5. The Effect of mTOR Kinase Inhibitors on Wound Healing and Kinetic Motility Assays

**Figure 5:**
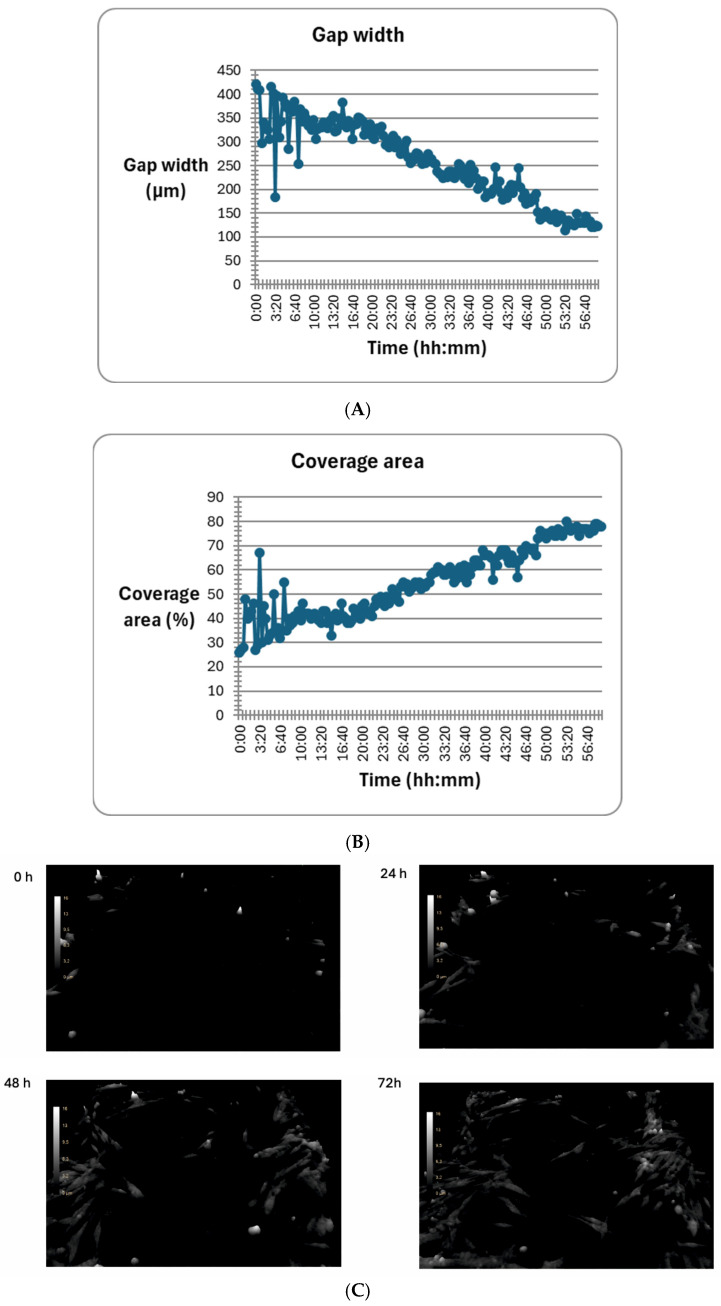
The effect of mTOR inhibitors on the In-depth Analysis: Wound Healing Assay: (**A**) gap width; (**B**) coverage area; (**C**) representative wound healing assay in MEWO melanoma cell line. The experiment was conducted for 60 (**A**,**B**) or 72 h (**C**), and 178 consecutive time points were measured in 6 independent biological replicates.

**Figure 6:**
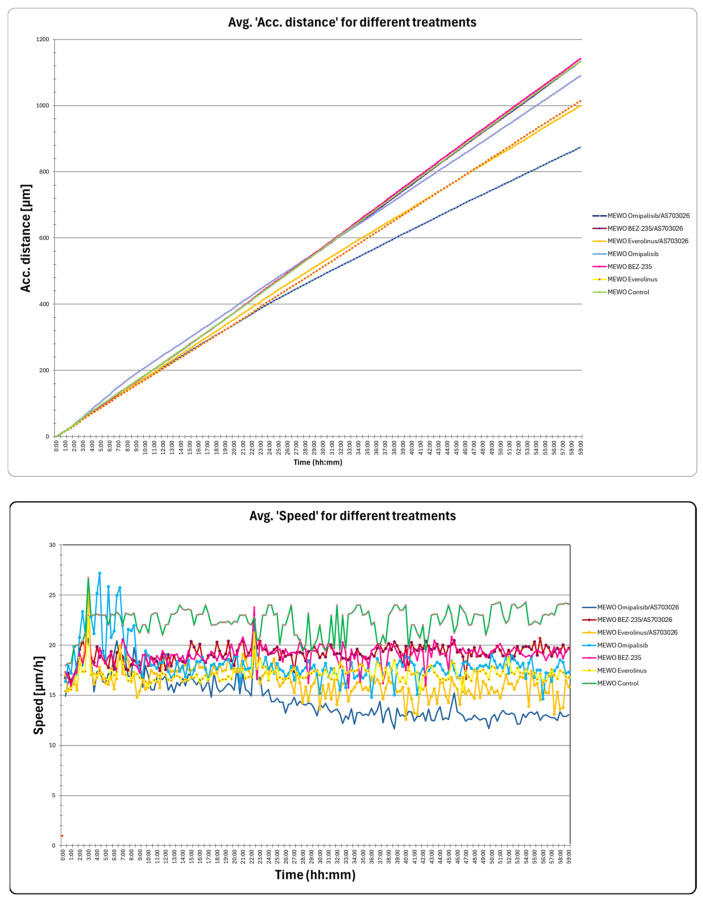
The effect of mTOR inhibitors on kinetic motility assays: accumulated mean cell distance; mean cell speed in MEWO melanoma cell line. The experiment was conducted for 60 h. For each inhibitor concentration tested, 178 consecutive time points were measured in 6 independent biological replicates.

We tested wound healing and kinetic motility assay for the MEWO melanoma cell line with the highest invasive potential (Figure 5 and Figure 6).

Figure 5 shows the dynamics of the gap width and coverage area process. Additionally, the process is illustrated by holographic photos taken at time intervals of up to 72 h (Figure 5).

We observed a significant change in the distance and maximum speed of MEWO melanoma cells after treatment with mTOR and MEK1/2 kinase inhibitors (Figure 6). The most significant decreases in maximum speed and distance were observed after the use of Omipalisib and Everolimus inhibitors, and their combinations with the MEK1/2 kinase inhibitor AS703026 (Figure 6).

### 2.6. The Effect of mTOR Kinase Inhibitors on Gelatinolytic Activities of the Matrix Metalloproteinases (MMPs) MMP-2 and MMP-9

The activities of two metalloproteinases, MMP-2 and MMP-9, were studied in the primary WM3211 and metastatic Mel-1359 and MEWO melanoma cell lines after 24 h and 48 h treatment with mTOR inhibitors and their combinations.

**Figure 7:**
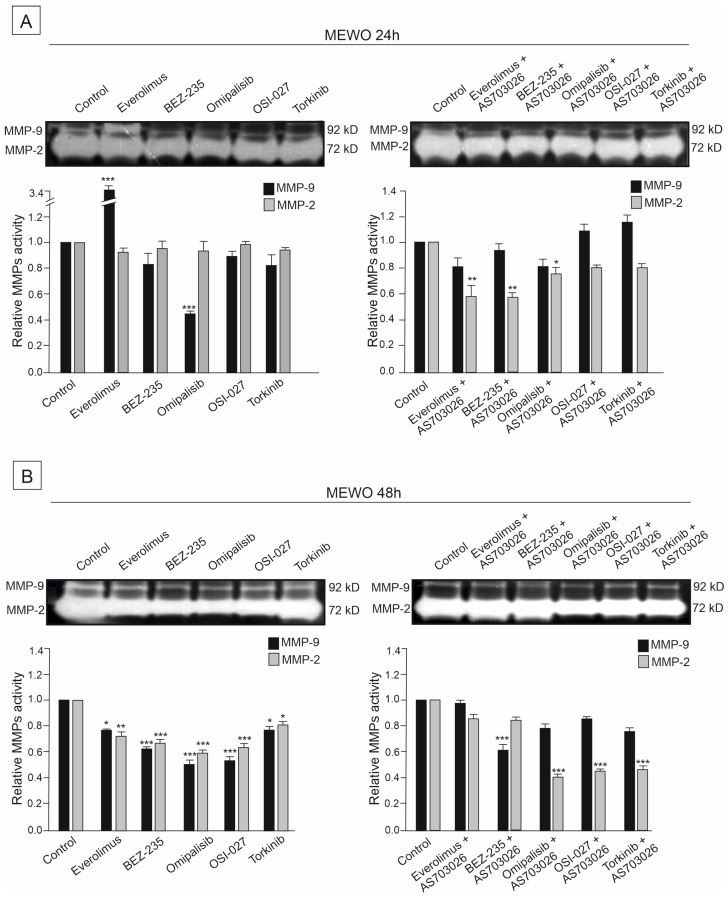
The effect of mTOR inhibitors (**A**) and the combination of mTOR inhibitors with the MEK1/2 inhibitor AS-703026 (**B**) on gelatinolytic activities of MMP-2 and MMP-9 in MEWO melanoma cell line. Statistical analyses of densitometric activity of MMP-2 and MMP-9 for at least three independent experiments with similar results were performed using one-way analysis of variance with Tukey’s post hoc test (Statistica 12.0 StatSoft); statistical significance at the level of (*) *p* < 0.05, (**) *p* < 0.01, (***) *p* < 0.001.

**Figure 8:**
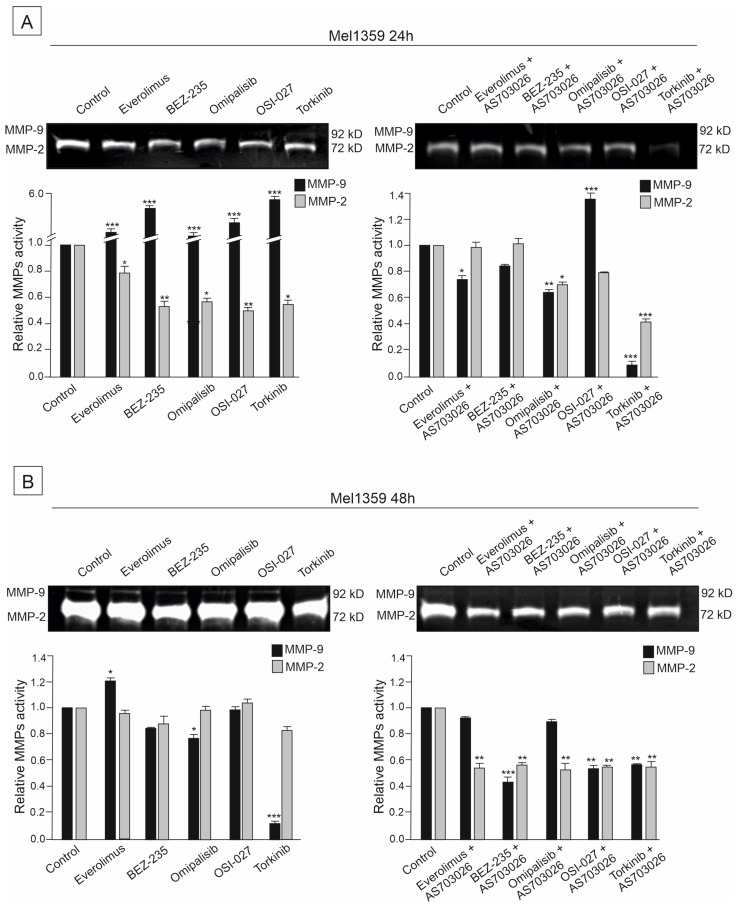
The effect of mTOR inhibitors (**A**) and the combination of mTOR inhibitors with the MEK1/2 inhibitor AS-703026 (**B**) on gelatinolytic activities of MMP-2 and MMP-9 in Mel-1359 melanoma cell line. Statistical analyses of densitometric activity of MMP-2 and MMP-9 for at least three independent experiments with similar results were performed using one-way analysis of variance with Tukey’s post hoc test (Statistica 12.0 StatSoft); statistical significance at the level of (*) *p* < 0.05, (**) *p* < 0.01, (***) *p* < 0.001.

**Figure 9:**
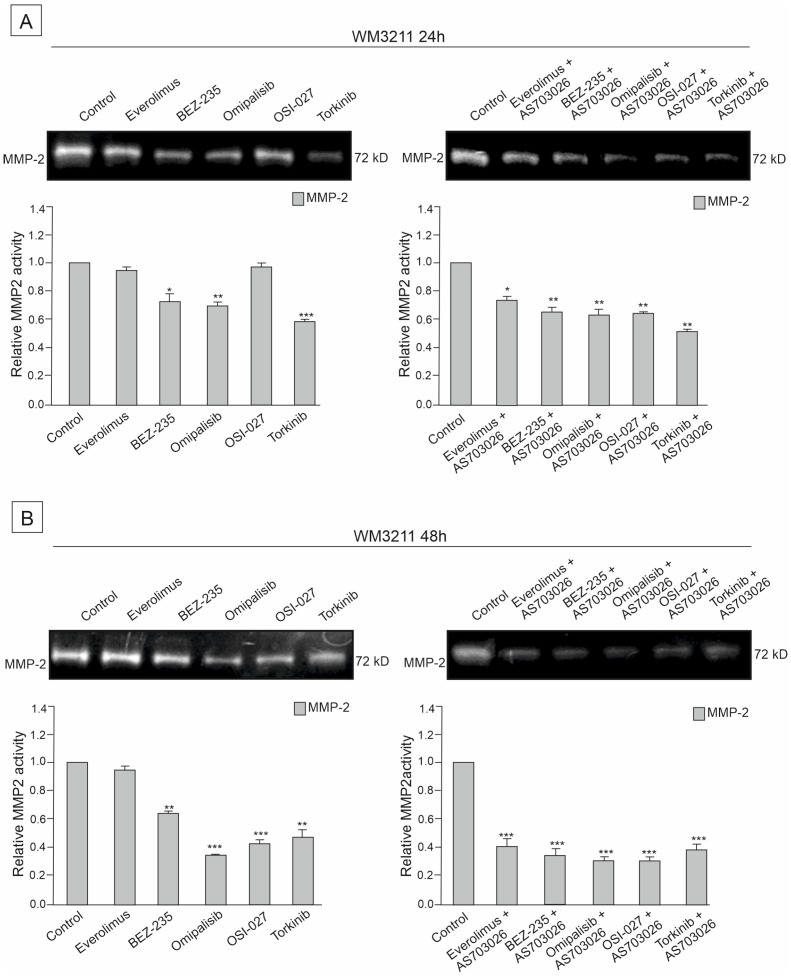
The effect of mTOR inhibitors (**A**) and the combination of mTOR inhibitors with the MEK1/2 inhibitor AS-703026 (**B**) on gelatinolytic activities of MMP-2 and MMP-9 in WM3211melanoma cell line. Statistical analyses of densitometric activity of MMP-2 and MMP-9 for at least three independent experiments with similar results were performed using one-way analysis of variance with Tukey’s post hoc test (Statistica 12.0 StatSoft); statistical significance at the level of (*) *p* < 0.05, (**) *p* < 0.01, (***) *p* < 0.001.

Metastasis-derived cell lines Mel-1359 and MEWO showed high levels of metalloproteinases; MMP-2 activity was high (Figure 7, Figure 8 and Figure 9). In the primary cell line WM3211, the level of metalloproteinases was significantly lower, and the expression of MMP-9 was not observed (Figure 7, Figure 8 and Figure 9 control lane).

The activity of metalloproteinase 2 in the MEWO cell line was very high, as was the activity of MMP- 9 (Figure 7 control line).

For 24 h, the use of mTOR kinase inhibitors in MEWO cell lines gave a slight effect of a decrease in metalloproteinase 2 activity. A similar trend was observed for MMP-9, except for the Everolimus inhibitor, for which an increase in activity was observed. Meanwhile, a significant decrease of approximately 50% (*p* < 0.001) in the activity of MMP-9 was observed after the use of the inhibitor Omipalisib (Figure 7A).

Using the combination of mTOR inhibitor with the MEK1/2 inhibitor AS-703026 was more effective than using single inhibitors. Decreases in the activity of MMP-2 reached up to 50% (*p* < 0.01), and, similarly to the use of single inhibitors, they concerned dual PI3K and mTOR inhibitors Omipalisib and Everolimus (Figure 7A).

For 48 h, the use of mTOR kinase inhibitors in MEWO cell lines gave a more pronounced effect of a decrease in metalloproteases than for 24 h (Figure 7B). Decreases in both metalloproteinases were observed, showing statistical significance (Figure 7B). The largest decreases in MMP-2 activity, ranging from 40 to 50% (*p* < 0.001), were observed after the use of BEZ-235, Omipalisib, and OSI-027 inhibitors (Figure 7B). After using the combination with the MEK1/2 AS-703026 inhibitor for the inhibitors Omipalisib, OSI-027, and Torkinib, a decrease in metalloproteinase activity of up to 55% was observed (*p* < 0.001) (Figure 7B).

A similar relationship was observed for the second metastatic melanoma cell line, Mel-1359, but the activity of metalloproteinases was significantly lower than for the MEWO cell line (Figure 7, Figure 8 and Figure 9). At 24 h, decreases in MMP-2 activity of 50% were observed for the inhibitors BEZ-235 (*p* < 0.01), Omipalisib (*p* < 0.5), OSI-027 (*p* < 0.01), and Torkinib (*p* < 0.5) (Figure 8A). On the other hand, the activity of MM-9 (which was significantly lower than that of the MEWO line) was higher than that of the control line (Figure 8A). For the use of a combination of mTOR inhibitors with a MEK1/2 AS-703026 inhibitor, the best effect was obtained for Omipalisib (about 40% (*p* < 0.01)) and Torkinib (about 50% (*p* < 0.001)) (Figure 8A).

For 48 h, the use of a combination of mTOR inhibitors with the MEK1/2 AS inhibitor gave an apparent effect of 50–55% for all the inhibitors used (*p* < 0.01) (Figure 8B).

The primary melanoma line WM3211 had an approximately 8 times lower level of MMP-2 than the MEWO line, and MMP-9 expression was not observed in the control sample (Figure 9).

The most significant effect of a decrease in MMP-2 activity by about 60% (*p* < 0.001) was obtained in the 48 h treatment with the combination with MEK1/2 AS-703026 inhibitors for all mTOR kinase inhibitors used (Figure 9B).

### 2.7. Effect of mTOR Inhibitors on the Expression of Cytoskeletal and Extracellular Matrix Proteins

In this study, we assessed the impact of three generations of mTOR kinase inhibitors, administered both individually and in combination with the MEK1/2 inhibitor AS-703026, on the expression of key adhesion and structural proteins in MEWO melanoma cells. The proteins evaluated included the cell adhesion molecules N-cadherin and E-cadherin, the cytoskeletal marker vimentin, and the extracellular matrix (ECM) components laminin (LAMC1) and fibronectin.

**Figure 10:**
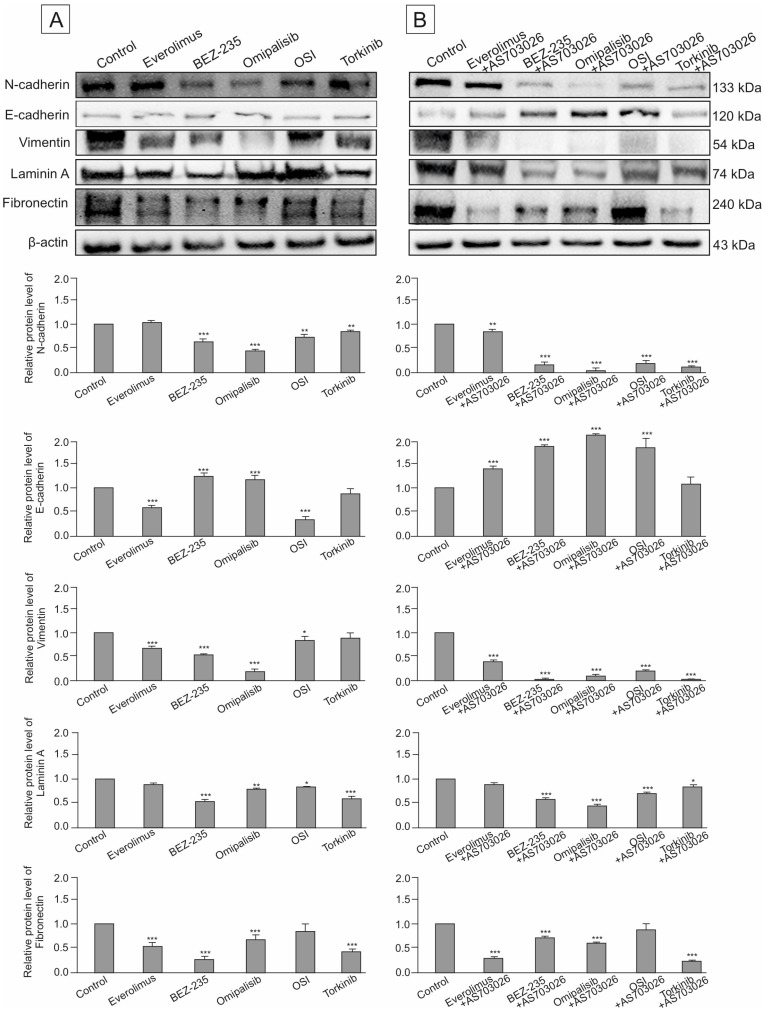
The effect of mTOR inhibitors (**A**) and the combination of mTOR inhibitors with the MEK1/2 inhibitor AS-703026 on the expression of N-cadherin and cytoskeletal and extracellular matrix proteins in MEWO melanoma cell lines. Actin was used as an internal loading control. (**B**) Densitometric quantification of protein expression was normalized to the corresponding β-actin levels. Presented values correspond to mean ± SD obtained from triplicate experiments. Statistical evaluation was conducted using one-way ANOVA followed by Dunnett’s post hoc test (Statistica 12.0, StatSoft). Statistically significant differences relating to the control group are denoted as follows: * *p* < 0.05, ** *p* < 0.01, *** *p* < 0.001.

Baseline expression levels of N-cadherin and other cytoskeletal/ECM proteins were notably high in the untreated control group (Figure 10A,B). Upon treatment, dual PI3K/mTOR inhibitors demonstrated the most substantial downregulation of N-cadherin, with Omipalisib reducing its expression by approximately 50% (*p* < 0.001), and BEZ-235 by around 40% (*p* < 0.001). Other mTOR-targeting agents led to more moderate reductions in the 10–20% range. The addition of the MEK1/2 inhibitor AS-703026 significantly enhanced this effect across most treatment regimens, except for Everolimus.

The most marked suppression of N-cadherin—approximately 85% (*p* < 0.001)—was observed when Omipalisib was combined with AS-703026. Combinations involving BEZ-235 or Torkinib resulted in roughly 75% (*p* < 0.001) reduction, while the OSI-027–AS-703026 pair reduced expression by 65% (*p* < 0.001) (Figure 10A,B).

In contrast, E-cadherin showed low basal expression in control cells. Treatment with two PI3K/mTOR inhibitors led to a moderate increase in E-cadherin levels, about 30% (*p* < 0.001 (Figure 10A). Remarkably, the combination of mTOR inhibitors BEZ-235 and Omipalisib with AS-703026 induced a statistically significant synergistic increase in E-cadherin expression by about 80% (*p* < 0.001). A slightly lower effect was observed for OSI inhibitors and Everolimus (Figure 10B). Regarding cytoskeletal remodeling, all mTOR inhibitors reduced vimentin expression to varying degrees. OSI-027 and Torkinib showed only limited efficacy. Omipalisib was the most effective single agent, decreasing vimentin expression by approximately 80% (*p* < 0.001). BEZ-235 and Everolimus reduced expression levels by 50–60% (Figure 10A,B).

Co-administration with AS-703026 amplified the anti-vimentin effect of several mTOR inhibitors, with dual PI3K/mTOR inhibitors (BEZ-235 and Omipalisib) reducing expression by up to 95% (*p* < 0.001). This indicates a strong synergistic suppression of cytoskeletal reorganization, a hallmark of invasive phenotype attenuation.

We also evaluated the expression of ECM proteins, including laminin (LAMC1) and fibronectin, both of which were abundantly expressed in the MEWO melanoma cells. BEZ-235 as a monotherapy reduced laminin levels by approximately 50% (*p* < 0.001). Combination therapies that included AS-703026 further potentiated this effect in all cases except OSI-027, which did not affect laminin expression.

The greatest reduction—up to 75% (*p* < 0.001)—was seen with the Torkinib and AS-703026 combination. In terms of fibronectin regulation, OSI-027 again demonstrated negligible impact, both as monotherapy and in combination. Conversely, Everolimus, BEZ-235, and Torkinib each produced robust downregulation of fibronectin (~60–65%, *p* < 0.001), an effect that was further enhanced upon MEK1/2 inhibitor co-treatment.

Collectively, these findings demonstrate that dual inhibition of the PI3K/mTOR and MEK pathways results in significant suppression of markers associated with cell adhesion, motility, and extracellular matrix remodeling, underscoring the potential of combination therapy strategies in mitigating the invasive behavior of melanoma cells.

## 3. Discussion

### 3.1. Modulation of Cell Morphology and Protein Expression by mTOR Pathway Inhibitors in Melanoma Cells

mTOR (mammalian target of rapamycin) is a serine/threonine kinase that regulates proliferation, growth, survival, and cytoskeletal organization. Its functional complexes—mTORC1, mTORC2, and mTORC3—play a key role in tumor progression, with mTORC1 and mTORC2 being particularly implicated in cancer development through modulation of cytoskeletal metabolism and dynamics [13]. Aberrant activation of this pathway has been observed in melanoma and various other malignancies.

In our study, we evaluated the effect of mTOR inhibitors, alone and in combination with the MEK1/2 inhibitor AS-703026, on MEWO melanoma cell morphology and expression of EMT-related proteins. The key innovation of this work is the integration of quantitative morphological analysis with cell vital processes to assess treatment response, which has been limited in previous studies. Morphological analysis revealed significant changes after treatment, including decreased cell thickness, altered shape, increased surface roughness, and decreased volume. These changes were most evident after treatment with dual PI3K/mTOR inhibitors (Omipalisib and BEZ-235), especially in combination with AS-703026. Such morphological remodeling may correspond to phenotypic shifts between mesenchymal and epithelial states and reflect underlying changes in cytoskeletal dynamics and adhesion properties [20]. Similar morphological abnormalities have been reported in other studies analyzing 3D melanoma cultures and single-cell imaging, confirming that surface texture and geometry reflect the invasive phenotype [20,21]. Such remodeling suggests a disrupted cytoskeletal organization and a shift toward a less invasive phenotype. These results are consistent with the suppression of EMT and induction of apoptosis, as previously reported in our work [22]. Additionally, a comparison with SK-MEL-28 and normal melanocytes showed that malignant cells exhibited greater shape variability and optical thickness [19].

These results are also in line with previous optical profiling studies using quantitative phase imaging, which demonstrated that melanoma cells show distinct morphological and optical signatures compared to normal melanocytes, supporting their utility as diagnostic and treatment response markers [19].

Dual PI3K/mTOR inhibitors reduced N-cadherin expression by 40–50%, and up to 85% in combination therapies (*p* < 0.001) [9,10]. In parallel, E-cadherin levels increased, suggesting EMT reversal. Vimentin, another EMT marker, was reduced by ~80% with Omipalisib and >90% when combined with AS-703026 (*p* < 0.001), consistent with the literature linking vimentin with metastatic potential [23,24,25,26]. Beyond its role as a marker, vimentin directly contributes to cell plasticity by modulating cytoskeletal stiffness, intracellular tension, and nuclear architecture, which influences migration efficiency and resistance to stress [25]. Laminin (LAMC1) and fibronectin, two ECM-related proteins associated with EMT and cell migration, were also suppressed, particularly with dual inhibitors (up to 60% and 65%, respectively), and this effect was synergistically enhanced by co-inhibition of MEK1/2. These ECM components are known to promote migration and invasion and are regulated by the PI3K/AKT/mTOR axis. The results of studies by Fang et al. and Peng et al. further support the role of these molecules by promoting EMT via TGFβ and AKT signaling [27,28].

These morphological alterations are closely associated with the observed inhibition of cell invasion, suggesting that reduced invasiveness may result from, or be reflected by, the remodeling of cytoskeletal and membrane architecture. Recent data also support that environmental and intracellular factors jointly regulate 3D morphology, which in turn influences motility and phenotypic plasticity in cancer cells [20].

Importantly, we also observed distinct changes in cellular morphology using fluorescence microscopy with acridine orange and DAPI staining. Treatment with mTOR and PI3K/mTOR inhibitors induced punctate orange fluorescence in the cytoplasm, indicative of acidic vesicular organelles such as lysosomes or autophagosomes, and reflecting autophagy activation. Concurrently, DAPI staining revealed chromatin condensation and nuclear fragmentation, hallmark features of apoptosis. These effects were significantly intensified in cells treated with combined mTOR and MEK inhibition, particularly with Omipalisib or BEZ-235 in combination with AS-703026. This dual staining approach confirmed the coexistence of apoptotic and autophagy processes in melanoma cells, underscoring a synergistic cytotoxic mechanism induced by the combination therapy. Thus, fluorescence-based imaging of nuclear and cytoplasmic changes complements quantitative phase imaging and molecular assays, providing an integrated view of drug-induced phenotypic remodeling.

A novel and clinically relevant aspect of this study is the identification of treatment-induced changes in cell morphology as potential surrogate markers of therapeutic efficacy. While previous studies have mainly focused on molecular markers of response, our results suggest that cellular morphological features, analyzed by label-free methods, may provide complementary diagnostic and predictive information. For example, morphological analysis of melanoma cells by holographic microscopy revealed differences in optical and morphological features between melanocytes and melanoma cells, which can be used to identify potential diagnostic and therapeutic markers [19].

Moreover, our findings align with updated models of tumor biology, which emphasize not only molecular mutations but also physical and structural features as integral hallmarks of cancer [2].

Combination therapy was significantly more effective than monotherapy. This may be due to the simultaneous blockade of two key signaling cascades: the PI3K/mTOR axis, which regulates growth, metabolism, and cytoskeletal stability; and the MEK/ERK pathway, responsible for proliferation and survival. Their parallel inhibition interrupts the compensatory feedback loops often activated during targeted monotherapy. Studies by Nardou et al. (2022) [29] confirm that co-inhibition of the mTOR and MEK pathways leads to synergistic effects, including enhanced inhibition of migration, induction of apoptosis, and greater downregulation of EMT markers [23,29]. This study provides the first integrated assessment of the effects of mTOR and MEK1/2 inhibitors on molecular markers of EMT and dynamic morphological features in a highly invasive melanoma model. Our findings demonstrate that mTOR and MEK1/2 inhibitors not only downregulate the expression of key EMT and ECM proteins but also significantly alter cell morphology in a manner consistent with reduced invasiveness. These results highlight the potential of targeting mTOR and MEK signaling pathways to impair melanoma progression by modulating cytoskeletal and membrane architecture [28].

### 3.2. Effect of mTOR Protein Kinase Inhibitors on Invasion and Metalloproteinase Activity in Melanoma Cells

The melanoma cell line MEWO, characterized by aggressive behavior and high MMP-2 activity, was used as a model to evaluate the effect of mTOR pathway inhibitors on cell invasion. In contrast, the primary melanoma cell line WM3211 showed significantly lower invasive potential and metalloproteinase expression.

Treatment with the dual PI3K/mTOR inhibitors BEZ-235 and Omipalisib resulted in the most significant reductions in invasion. These effects were further enhanced when combined with the MEK1/2 inhibitor AS-703026, resulting in up to 50% inhibition. Interestingly, a significant reduction in migration indices (distance and velocity) was observed after treatment with Omipalisib, Everolimus, and their combinations with AS-703026.

This anti-invasive effect correlates with the observed reduction in morphological complexity and surface irregularity (Section 3.1), supporting the concept that cytoskeletal remodeling and motility suppression are coupled at the molecular and phenotypic levels.

Previous studies confirm our observations. Chen et al. [23] showed that BEZ-235 inhibited proliferation in drug-resistant gastric cancer cells, while combination therapy with Regorafenib reduced invasiveness and MMP activity in hepatocellular carcinoma [30]. Similar effects were demonstrated in renal cancer, where BEZ-235 was superior to Rapamycin and Torkinib [31].

Moreover, BEZ-235 has been shown to inhibit PI3K/mTOR-dependent signaling nodes such as AKT, S6, and 4EBP1, reducing MMP-2 expression and invasive migration across multiple tumor types [31,32]. These findings align with our results in melanoma and support BEZ-235 broad anti-metastatic potential.

Our work adds new value by combining metalloproteinase inhibition with real-time functional behavior of melanoma cells under dual blockade conditions, providing a dynamic readout of therapeutic impact. BEZ-235, an orally bioavailable dual inhibitor, currently in clinical trials, has shown activity in a broad spectrum of malignancies, increasing drug sensitivity and overcoming resistance [32].

Omipalisib (GSK2126458) is another potent dual inhibitor targeting all class I PI3K isoforms and mTORC1 and mTORC2 complexes [33]. Preclinical studies have shown its ability to inhibit AKT, S6, and 4EBP1 phosphorylation, with broad antitumor efficacy. Phase I data support its potential in various solid tumors. Importantly, Omipalisib also inhibits MMP expression and ECM remodeling, possibly through suppression of NF-κB and downstream cytokine production [33], reinforcing its utility as an anti-invasive agent.

An important piece of information from our study is the synergistic effect of combining mTOR/PI3K inhibitors with MEK1/2 blockades, especially in NRAS-mutated melanoma, where reciprocal activation limits the efficacy of monotherapy. This is consistent with the results of Nardou et al. [29], who showed that Omipalisib combined with MEK inhibition significantly reduced tumor growth in xenograft models and induced synergistic apoptosis in vitro. Among the MEK inhibitors tested, AS-703026 was the most effective in activating apoptotic signaling in melanoma cells [29,34]. Our findings [35] reinforce the utility of combination therapy by demonstrating that co-treatment with Omipalisib and AS-703026 significantly activates apoptotic pathways while limiting invasive behavior.

This dual-targeting approach interferes with parallel and feedback-amplifying survival mechanisms, including AKT-mediated MMP activation and ERK-mediated cell motility signals, offering a mechanistically sound basis for combination therapy [28].

This dual effect on phenotypic and biochemical hallmarks of malignancy represents a key advance over previous studies focused solely on molecular endpoints. By integrating invasion assays with morphological and biochemical analyses, our model bridges the gap between phenotypic plasticity and mechanistic signaling events, offering a comprehensive evaluation of treatment efficacy.

In summary, this study highlights the enhanced efficacy of combined PI3K/mTOR and MEK inhibition in limiting invasion and metalloproteinase activity in melanoma. Integrating functional markers of cell motility with morphological profiling and molecular endpoints offers a more comprehensive framework for evaluating anticancer strategies and identifying effective drug combinations.

## 4. Materials and Methods

A summary of the methods used is provided in Table 2.

### 4.1. Cell Culture

Primary (VGP)—WM3211—and metastatic—MEWO and Mel 1359—human melanoma cell lines were used. The primary human melanoma cell line (VGP) was wild-type for BRAF, PTEN, N-RAS, and CDK4, and has a mutation in the c-KIT gene at position 576.

The MEWO human malignant melanoma cell line was derived from a lymph node and was wild-type for the BRAF, PTEN, and N-RAS genes. In contrast, the Mel-1359 human malignant melanoma cell line featured a mutation in the BRAF gene while remaining wild-type for both PTEN and N-RAS.

The cells were cultured in RPMI-1640 medium containing 10% fetal bovine serum and antibiotics such as penicillin and streptomycin. They were incubated at 37 °C in a humidified atmosphere with 5% CO_2_. The cells were treated with the following inhibitors: 1. MEK1/2 inhibitor—AS-703026 (Selleck) at a concentration of 10 μM; 2. mTOR inhibitor—Everolimus (Selleck) at a concentration of 20 nM; 3. dual PI3K and mTOR inhibitor—BEZ-235 (NVP-BEZ235, Selleck) at a concentration of 20 nM; 4. dual PI3K and mTOR inhibitor—Omipalisib (GSK2126458, Selleck) at a concentration of 20 nM; 5. mTOR1/2 inhibitor—OSI-027 (Selleck) at a concentration of 20 nM; and 6. mTOR inhibitor—Torkinib (PP242, Selleck) at a concentration of 20 nM. All kinase inhibitors were purchased from Selleck Chemicals (Houston, TX, USA).

Melanoma cells were incubated with these inhibitors for 24 or 48 h.

This manuscript describes established melanoma cell lines derived from the ESTDAB (University of Tübingen Centre for Medical Research, ZMF, Waldhörnlestr. 22, 72072 Tubingen, Germany), in which we participated by characterizing melanoma cell lines for the ESTDAB database (https://www.ebi.ac.uk/ipd/estdab/ accessed on 1 January 2006). Synonyms: WM-3211; WM 3211; WC00045; EST79, ESTDAB-079; Synonyms: MEWO; Mewo; Me Wo; Me-Wo; Mevo; SK-MEL-MeWo; Mel-MeWo; BI-Mel; EST50, ESTDAB-050; Synonyms: Mel 1359; 1359-MEL; 1359 mel; 1359; Mel-1359; ESTDAB-047.

### 4.2. Holographic Microscopy

The studies were conducted using Phase Holographic Imaging (PHI AB, Lund, Sweden), and the experiments took place in a CO_2_ cell culture incubator. Melanoma cells were cultured in 24-well plates (Sarstedt, Nümbrecht, Germany) and covered with HoloLids (PHI, Lund, Sweden) to maintain stable imaging conditions. The melanoma cells received treatment with mTOR and MAP kinase inhibitors.

The experiment was conducted over a period of 72 h, calibrating six imaging positions per well. Time points were taken every 20 min at 37 °C and 5% CO_2_. For each inhibitor concentration tested, 178 consecutive time points were measured in 6 independent biological replicates, with the experiment lasting either 72 or 60 h.

### 4.3. Quantitative Analysis of Cell Morphology and Motility

The In-depth Analysis: “Cell Morphology” module of the Holomonitor App Suite (PHI, Sweden) was used to track the movement of single cells. The following cell identification settings were used: automatic minimum error method, 134 adjustments, and minimum object size 20. Measurements of optical thickness, optical volume, roughness, shape convexity, irregularity, and motility were performed at specific time points. Using the “Kinetic Assays” module: “Kinetic Motility Assays”, In-depth Analysis: “Wound Healing Assay” was studied.

### 4.4. Fluorescence Microscopy: Acridine Orange and DAPI Staining in Transmitted Light

Fluorescent staining was performed to assess nuclear and cytoplasmic structures in MEWO melanoma cells using acridine orange (AO APEx BIO, Houston, TX, USA) and 4′,6-diamidine-2′-phenylindole dihydrochloride (DAPI, Roche, Basel, Switzerland). Melanoma cells were stained with DAPI (4′,6-diamidino-2-phenylindole dihydrochloride; Roche, Basel, Switzerland) according to the procedure outlined in the Roche protocol (Cat. No. 10 236 276 001). Acridine orange and propidium iodide co-staining was carried out using Kit K2238 (APEx BIO, Houston, TX, USA), according to the manufacturer’s recommended protocol.

Fluorescent imaging of melanoma cells was performed using an Olympus fluorescence microscope (Tokyo, Japan).

### 4.5. Cell Invasion Assay

Cell invasion assays were performed using conventional Boyden trans-well methods, maintaining the manufacturer’s protocol (BD BioCoatTM FluoroBlok Invasion System No. 354166, BD Biosciences, Franklin Lakes, NJ, USA) described previously [36,37]. Melanoma cell lines WM3211, MEWO, and Mel1359 in serum-free medium in a 24-well invasion plate at a concentration of 5 × 10^4^/mL were placed in the upper part of a Boyden chamber, and a medium with 10% serum was added to the lower part as a chemoattractant. After 24 h, fluorescence of invasive cells was measured, previously stained with Calcein AM (Thermo Fisher Scientific, Waltham, MA, USA). All melanoma cells examined showed the ability to migrate (chambers without matrigel).

### 4.6. Zymography

Preparation of samples for gelatin zymography and densitometry analyses of gelatinolytic activities of metalloproteinases: MMP-2 and MMP-9 described previously [36]. Melanoma cell lines WM3211, MEWO, and Mel1359 were cultured in serum-free medium (5 × 10^4^/mL) in a 24-well plate two different times (24 h and 48 h). In serum-free medium, the activities of metalloproteinases MMP-2 and MMP-9 were analyzed using gelatin zymography.

### 4.7. Cytotoxicity Assay

Cytotoxicity of selected kinase inhibitors (MEK1/2—AS-703026 (10 μM); mTOR– Everolimus (20 nM); dual PI3K and mTOR inhibitor—BEZ-235 (20 nM); dual PI3K and mTOR inhibitor—Omipalisib (20 nM); mTOR1/2– OSI-027 (20 nM); mTOR– Torkinib (20 nM); and DMSO) was determined using Cytotoxicity Detection Kit LDH (Roche, Roche, Basel, Switzerland). mTOR and MEK kinase inhibitors were prepared as stock solutions in DMSO at a concentration 1000 times greater than that used in cell culture (dilution: 1 µL of stock solution into 1 mL of cell culture medium). We tested the inhibitors used for cytotoxicity for melanoma cells cultured both in RPMI-1640 medium containing 10% fetal bovine serum and without serum. All kinase inhibitors were purchased from Selleck Chemicals (Houston, TX, USA).

### 4.8. Western Blot Analysis

The procedure for sample preparation used in SDS-PAGE electrophoresis was performed according to a previously described protocol [36]. The following primary antibodies were employed to detect target proteins: mouse anti-N-cadherin (610920, BD Biosciences, Franklin Lakes, NJ, USA), mouse anti-E-cadherin (C20820, BD Biosciences, Franklin Lakes, NJ, USA), mouse anti-fibronectin (610077, BD Biosciences, Franklin Lakes, NJ, USA), rabbit anti-vimentin (A19607-20, ABclonal, Wuhan, China), rabbit anti-laminin subunit gamma-1 (LAMC1, A16020, ABclonal Wuhan, China), and mouse anti-β-actin (A2228, Sigma-Aldrich St. Louis, MO, USA), which served as the internal loading control.

Protein bands were visualized using horseradish peroxidase (HRP)-conjugated secondary antibodies specific to mouse or rabbit IgG (Cell Signaling Technology, Danvers, MA, USA), followed by detection via enhanced chemiluminescence. Signal acquisition was performed using the ChemiDoc MP Imaging System (Bio-Rad Laboratories, Hercules, CA, USA). Quantitative analysis of band intensity was conducted using SynGene Gene Tools software, version 4.03.0 (Synoptics Ltd., Cambridge, UK). Densitometric values were normalized against β-actin to account for loading variations. Representative blots from a minimum of three independent biological replicates, yielding consistent results, are shown in the figures.

### 4.9. Statistics

Invasion measurements represent mean values calculated from multiple independent experiments. Statistical analyses were performed using one-way ANOVA with post hoc Tukey test (Statistica 12.0 StatSoft); statistical significance at the level: (*) *p* < 0.05, (**) *p* < 0.01, (***) *p* < 0.001.

## 5. Conclusions

Our findings demonstrate that mTOR kinase inhibitors from three successive generations, particularly when used in combination with MEK1/2 inhibition, significantly reduce melanoma cell invasiveness and downregulate metalloproteinases MMP-2 and MMP-9. These results, together with previously reported effects on apoptosis induction and proliferation suppression, highlight the therapeutic potential of dual-targeted approaches.

Crucially, our findings underscore the diagnostic and prognostic value of real-time, label-free holographic microscopy for monitoring dynamic changes in cell morphology. Quantitative features such as thickness, volume, shape irregularity, and surface roughness turned out to be highly sensitive indicators of treatment response. Holographic profiling provides a robust, non-invasive platform for tracking phenotypic shifts during targeted therapy and may serve as a valuable clinical tool to support personalized treatment strategies in malignant melanoma.

## Figures and Tables

**Table 1:** Summary of the effects of mTOR inhibitors on cell morphology. For each inhibitor concentration tested, 178 consecutive time points were measured in 6 independent biological replicates. Statistical comparisons were performed using one-way ANOVA followed by Tukey’s post hoc test (Statistica version 12, StatSoft). Levels of statistical significance are de-noted as follows: (*) *p* < 0.05; (**) *p* < 0.01; (***) *p* < 0.001. Yellow indicates a decrease, and blue indicates an increase in the feature.

	Inhibitor	Optical Thickness (µm)	Optical Volume (µm^3^)	Roughness Avg	Shape Convexity	Irregularity
1	Everolimus	*	*	*	*	*
2	BEZ-235	*	**	*	**	**
3	Omipalisib	*	**	*	*	**
4	Everolimus/ AS-703026	**	**	**	*	**
5	BEZ-235/ AS-703026	**	***	**	**	***
6	Omipalisib/ AS-703026	***	***	***	***	***

**Table 2:** Experimental design summary.

Parameter	Details
Cell lines	MEWO and Mel-1359 (metastatic), WM3211 (primary)
Inhibitors used	Everolimus (mTORC1 inhibitor) Torkinib, OSI-027 (mTORC1/2 inhibitors) BEZ-235, Omipalisib (dual PI3K/mTOR inhibitors) AS-703026 (MEK1/2 inhibitor)
Concentrations	AS-703026—10 µM mTOR inhibitors—20 nM
Controls	DMSO (≤0.1–2%)—no cytotoxic effect observed under any condition
Culture conditions	With 10% FBS: Used in all experiments except invasion and zymography Without FBS: Used only in zymography (MMP assay) and Boyden invasion assay
Incubation times	24 h: Western blot (protein expression), zymography (MMP activity), invasion assay 48 h: Extended zymography (MMP) 60–72 h: Cell morphology (holography), cytotoxicity, wound healing
Assays performed	Serum-free: Gelatin zymography (MMP-2, MMP-9), trans-well invasion assay, cytotoxicity assay With serum: Western blot, morphology analysis, cytotoxicity, wound healing assay
Replicates	≥3 independent biological replicates; ≥4 technical replicates per condition

## Data Availability

No datasets were generated or analyzed during the current study.
